# Multiple Sampling in One Day to Optimize Smear Microscopy in Children with Tuberculosis in Yemen

**DOI:** 10.1371/journal.pone.0005140

**Published:** 2009-04-09

**Authors:** Nasher Al-Aghbari, Najla Al-Sonboli, Mohammed A. Yassin, John B. S. Coulter, Zayed Atef, Ali Al-Eryani, Luis E. Cuevas

**Affiliations:** 1 National Tuberculosis Institute, Sana'a, Yemen; 2 Medical Faculty, Sana'a University, Sana'a, Yemen; 3 Liverpool School of Tropical Medicine, Liverpool, United Kingdom; McGill University, Canada

## Abstract

**Background and Aim:**

The diagnosis of pulmonary Tuberculosis (TB) in children is difficult and often requires hospitalization. We explored whether the yield of specimens collected for smear microscopy from different anatomical sites in one visit is comparable to the yield of specimens collected from a single anatomical site over several days.

**Methodology and Principal Findings:**

Children with signs/symptoms of pulmonary TB attending a reference hospital in Sana'a Yemen underwent one nasopharyngeal aspirate (NPA) the first day of consultation and three gastric aspirates (GA) plus three expectorated/induced sputa over 3 consecutive days. Specimens were examined using smear microscopy (Ziehl-Neelsen) and cultured in solid media (Ogawa). Two hundred and thirteen children (aged 2 months–15 years) were enrolled. One hundred and ninety seven (93%) underwent nasopharyngeal aspirates, 196 (92%) GA, 122 (57%) expectorated sputum and 88 induced sputum. A total 1309 specimens were collected requiring 237 hospitalization days. In total, 29 (13.6%) children were confirmed by culture and 18 (8.5%) by smear microscopy. The NPA identified 10 of the 18 smear-positives; three consecutive GA identified 10 and induced/expectorated sputa identified 13 (6 by induced, 8 by expectorated sputum and one positive by both). In comparison, 22 (3.7%) of 602 specimens obtained the first day were smear-positive and identified 14 (6.6%) smear-positive children.

**Conclusion/Significance:**

The examination of multiple tests the first day of consultation identified a similar proportion of smear-positive children than specimens collected over several days; would require half the number of tests and significantly less hospitalization. Optimized smear microscopy approaches for children should be explored further.

## Introduction

The microbiological confirmation of pulmonary Tuberculosis (PTB) in children is difficult [1]. Although the diagnosis of PTB in older children and adults in low and middle income countries is based on the examination of serial sputum specimens using direct smear microscopy, children under 5 years old are often unable to expectorate sputum and have paucibacillary disease presentation and other methods are therefore needed to obtain specimens [2], [3].

The examination of gastric aspirate (GA), nasopharyngeal aspirates (NPA) and of induced sputum have been reported to be useful in children [4]–[6]. These methods however often have a low yield and are onerous to the parents as GA requires the hospitalization of the child to obtain specimens for several days and approaches that reduce the number of days to complete the diagnosis are necessary. Diagnostic methods are rarely compared to explore if their use in combination could result in increased sensitivity or whether more efficient screening approaches that required less time to complete the investigations could be developed.

We report here the additional yield obtained by using a combination of nasopharyngeal aspirates (NPA), gastric aspirate (GA) plus induced and expectorated sputum in children with a clinical suspicion of PTB, and describe whether the combination of samples collected the first day of consultation could result in a similar yield than samples obtained by any of the techniques over three consecutive days.

## Methods

### Ethics statements

The study was approved by the research ethics committees of the National TB Centre in Sana'a and the Liverpool School of Tropical Medicine, Liverpool, UK. Oral consent was obtained from the parents/guardians of the children before inclusion in the study. Oral consent, in the presence of a witness, is the routine approach used in this hospital setting. Parents are often reluctant to sign papers and the ethics committees agreed that oral consent was acceptable in these circumstances.

Children less than 15 years old attending Althawra and Alsabeen hospitals and the National Tuberculosis Centre (NTC) in Sana'a, Yemen from October 2003 to June 2005 with a clinical suspicion of PTB were invited to participate. These health centers act as referral centers for the region and serve a population of about 2 million. Children were deemed eligible for the study if they had any of the World Health Organization (WHO) criteria for *suspected* TB in children. These included a history of contact with cases of PTB, children not regaining normal health after measles or whooping cough, unexplained weight loss, the presence of cough and wheeze not responding to antibiotic therapy. Children who had X-ray findings suggestive of (PTB) were also included [7]. According to the National TB Control Programme (NTCP), there are 82 cases of TB (all forms) in every 100,000 population. There are however no reliable statistics on the number of paediatric TB cases in the country.

Clinical specimens for examination included one NPA, three GA and three expectorated or three induced sputum specimens. NPAs were obtained by direct aspiration via a mucus trap connected to a suction device on the first day, without induction of cough or instillation of saline solutions. Specimens were transferred into 2 ml sterile cryotubes after the addition of 2 ml of phosphate buffered saline (PBS) and vortex mixing.

Gastric aspirates were obtained at 6:00 am on three consecutive days by introducing a nasogastric tube and aspiration of the gastric content with a syringe. Aspirates were transferred into sterile containers and an equal volume of PBS (pH = 7.0) was added to maintain the viability of the bacilli [4], [5].

Children were also asked to expectorate sputum and, if unattainable, underwent induced sputum. Induction was obtained using inhaled salbutamol via a metered dose inhaler attached to oxygen at a flow rate of 5 liters per minute on 5 ml of 5% sterile saline for 15 minutes [8], [9]. This was followed by physiotherapy (chest percussion, vibration and active cycle breathing) and sputum collection by expectoration or from the naso/orophrarynx using a mucus extractor in those unable to expectorate.

### Laboratory methods

After the preparation of direct smears, specimens were stained using the hot Ziehl Neelsen (ZN) method. All smears were read and graded by trained microscopists at the NTC who were unaware of the grading of the previous specimens. All specimens were cultured using Ogawa culture media and positive cultures were examined by smear microscopy of the culture isolates to confirm the presence of acid-fast bacilli (AFB) and by the niacin test.

The data were entered into computer databases and analyzed using EPI-Info 2000 (CDC, Atlanta, USA). The total yield obtained by examining all specimens and those collected using a single technique over three days were calculated. These yields were compared to the total yield obtained by examining all specimens obtained from a child the first day of investigation using descriptive statistics.

## Results

Two hundred and thirteen children with a clinical suspicion of PTB were included. Their age ranged from 2 months to 15 years, with a median of 5 years and 42 (20%) were <2 years old. Seventy nine (37%) children were hospitalized for diagnosis and 134 (63%) were investigated while ambulatory. The most frequent clinical symptoms at presentation were cough (195, 92%), unexplained fever (179, 84%), anorexia (142, 67%), weight loss (125, 59%) and difficult breathing (82, 38%). One hundred and nine (51%) children had a relative at home with a diagnosis of TB and a further 14 indicated having a relative with chronic cough.

Investigations included nasopharyngeal aspirates in 197 (92%) children, gastric aspirates in 196 (92%), spontaneous sputum in 122 (57%) and induced sputum in 88 (41%). The number of tests performed each day is shown in table 1 and 2. The proportion of single smears being positive each day was low, with a range of between 2% and 7% for any of the collection techniques used. Some children had positive smears on consecutive days and the cumulative yield of examining multiple specimens over three days ranged from 5% for gastric aspirates to 7% and 7% for induced and expectorated sputum, respectively. In total, 52 (4%) of the 1309 smears examined were positive, with 18 (8.5%) children having one or more positive smears and 29 children had one or more positive cultures. All children with positive smears had positive cultures.

**Table 1:** Proportion of smears positive and additional yield of specimens collected by expectorated or induced sputum and gastric and nasopharyngeal aspirates.

Specimen	Day 1	Day 2		Day 3		Total	
	Pos/N (%)	Pos/N (%)	Additional yield	Pos/N (%)	Additional yield	Pos/N (%)	Cumulative yield
Nasopharyngeal aspirate	10/200 (5%)	-	-	-	-	10/200 (5%)	10/200 (5%)
Gastric aspirate	5/203 (3%)	6/189 (3%)	3	6/172 (4%)	2	17/564 (3%)	10/203 (5%)
Expectorated sputum	5/117 (4%)	6/113 (5%)	2	5/99 (5%)	1	16/329 (5%)	8/117 (7%)
Induced sputum	2/82 (2%)	3/72 (4%)	1	4/62 (7%)	3	9/216 (4%)	6/82 (7%)
All specimens	22/602 (4%)	15/374 (4%)		15/333 (5%)		52/1309 (4%)	
Additional yield* N = 213	14 (6.5%)		2		2		18 (8.5%)

* Number of additional children identified, N = number, pos = positive.

**Table 2:** Proportion of positive cultures and additional yield of cultured specimens collected by expectorated or induced sputum and gastric and nasopharyngeal aspirates.

Specimen	Day 1	Day 2		Day 3		Total	
	Pos/N (%)	Pos/N (%)	Additional yield	Pos/N (%)	Additional yield	Pos/N (%)	Cumulative yield
Nasopharyngeal aspirate	14/200 (7%)	-	-	-	-	14/200 (7%)	14/200 (7%)
Gastric aspirate	18/203 (9%)	14/189 (7%)	1	17/172 (10%)	0	49/564 (9%)	19/203 (9%)
Expectorated sputum	9/117 (8%)	9/113 (8%)	5	9/99 (9%)	0	27/329 (8%)	14/117 (12%)
Induced sputum	12/82 (15%)	9/72 (13%)	1	10/62 (16%)	0	31/216 (14%)	13/82 (16%)
All samples	53/602 (9%)	32/374 (9%)		36/333 (11%)		121/1309 (9%)	
Additional yield* N = 213	29 (13.6%)		0		0		29 (13.6%)

* Number of additional children identified, N = number, pos = positive.

Among the specimens collected on the first day, induced sputum had the lowest (2%, 2/82) and NPA the highest (5%, 10/200) proportion of positive smears, although the difference was not statistically significant. In total, 22 (4%) of the 602 specimens obtained on the first day were smear positive, and these had been obtained from 14 children (table 1).

Although the combination of multiple smear tests over three consecutive days resulted in the highest yield of smear microscopy (18 children), this approach required examining 1309 specimens and 237 hospitalization days. The second highest yield was obtained by combining all the tests obtained in the first day (14 children, 78% of the smear-positive children), which would require half the number of tests and a much lower number of hospitalizations.

The proportion of positive cultures and the additional yield of the cultures of samples obtained by each technique are shown in table 2. The proportion of positive cultures ranged from 7% to 16% of the samples obtained by each technique. None of the sampling techniques seemed to have a higher yield than the others, although the cumulative yield of culture of expectorated and induced sputum was higher than the yields of GA and NPA. In total, 29 (13.5%) of the 213 children had at least one positive culture, but their identification required multiple sampling techniques, as a single technique was not able to identify all of them. In comparison, the cumulative yield of all the cultures obtained the first day of consultation resulted in the same yield of 29 children.

## Discussion

The World Health Organization has recently issued new guidelines for the diagnosis of TB in children and emphasized the importance of the bacteriological confirmation of diagnosis [3]. According to these guidelines, most children with TB have PTB and smear microscopy is a useful tool for confirmation of the diagnosis in older children in developing countries, where culture facilities are limited. However, even though sputum smear microscopy can be positive in the majority of adults with PTB, fewer than 20% of children with TB have a positive smear in sputum or gastric lavage [10]. Indeed, in this study neither sputum nor GA resulted in more than 10% positives.

The revised guidelines indicate that expectoration, gastric aspirate and sputum induction are the most frequently used methods to obtain specimens in children, although the guidelines do not specify whether to use these methods singly or in combination [3]. There are few studies that have explored whether the collection of several samples from different sites could be synergistic and increase the recovery of *Mycobacterium tuberculosis* among symptomatic children [6]. A number of studies in adults have reported that some techniques are more likely to yield positive smears than others or that the yield of relatively less invasive techniques, such as sputum induction, could be as good as the yield of more invasive techniques, such as fibreoptic bronchoscopy [11], [12] and is thus possible that different combinations of sampling techniques and number of specimens could be used to optimize smear microscopy in children.

The findings reported in this study demonstrate that using samples from multiple sites over consecutive days results in a higher additional yield (18, 8.5%) than obtaining multiple samples from a single site. This approach would require examining a very large number of specimens, increase the need for hospitalization and would not be suitable for children in high burden settings. Examining samples with a single technique over several days, as it is routinely used in most high burden settings identified between 6 and 10 of these patients, while an alternative approach of examining all specimens collected from multiple sites in a single day identified 14 of the children. This approach would require less diagnostic visits and hospitalizations with significant savings for the parents, and would be easier to where hospitalization of children is not feasible or where access to health services is limited.

Furthermore, culturing specimens obtained with several sampling techniques in a single day resulted in the identification of all children with positive cultures and culturing samples obtained in subsequent days did not result in further gains, reflecting the fact that culture is more sensitive than smear microscopy, regardless of the site of specimen collection.

The data presented here suggest that it is possible to develop new approaches that optimize the yield of smear microscopy in children in high incidence settings. These approaches need to consider that children have difficulty providing suitable specimens for smear microscopy and that their parents often need to travel at considerable expense to reach health facilities.

Although the study used consensus criteria from recent WHO guidelines for the diagnosis of TB in children; provided an unbiased reading of smears through the blinding of the microscopists to the results of previous specimens and the high percentage of children who underwent gastric and nasopharyngeal aspiration; the findings reported need to be further investigated, as the small sample size of the study prevented analysis stratified by age and young children may behave differently than older children. In addition, although the HIV status of the children was not available, the prevalence of TB and HIV co-infection among adults attending the TB Institute in Sana'a is low (<1%, unpublished), and the findings cannot be extrapolated to areas with high HIV prevalence. Despite these limitations, the findings reported here suggest that smear microscopy approaches could be optimized for children and these are urgently needed.
